# Effects of dietary mangosteen peel powder and extract on the growth performance, meat quality and indicators for immunity, gut health and antioxidant activity in broiler chicks

**DOI:** 10.1016/j.psj.2024.104477

**Published:** 2024-10-31

**Authors:** Da-Hye Kim, Hyeon Mo Yang, Ju-Yong Song, Jina Park, Byung-Yeon Kwon, Anh Viet Vu, Dae Sung Lee, Kyung-Woo Lee

**Affiliations:** a Department of Animal Science and Technology, Konkuk University, 120 Neungdong-ro, Gwangjin-gu, Seoul 05029, Republic of Korea; b Medi Bio Lab Co., Ltd., Seoul Republic of Korea

**Keywords:** Antioxidant activity, Growth performance, Mangosteen peel, Gut health, Broiler chickens

## Abstract

The objective of this study was to evaluate the effects of dietary mangosteen peel preparations, either powdered (MspP) or ethanolic extract (MspE), on the growth performance, meat quality, immune response, gut health, serum biochemical profiles, and antioxidant activity of broiler chicks. A total of 480 day-old straight-run broiler chicks (Ross 308) were randomly placed into four treatments, with eight replicates of 12 chicks each, and subjected to one of the four experimental diets for 21 days. The corn and soybean meal-based diet was supplemented with 2% MspP (20 g per kg of diet) or 0.05% and 0.1% MspE (0.5 g and 1.0 g per kg of diet). Data were analyzed using analysis of variance, and *post hoc* comparisons of treatments were performed using Tukey's Honestly Significant Difference test. From days 0 to 21, dietary mangosteen peel preparations did not affect growth performance (body weight gain, feed intake, and feed conversion ratio), thigh meat and tibia characteristics, serum markers of innate immunity (interferon-r, interleukin-10, alpha-1-acid glycoprotein, and nitric oxide), and ileal morphology in broiler chicks (*P* > 0.05). Dietary mangosteen peel preparations increased the percentage of high-density lipoprotein cholesterol and decreased the relative concentrations of isobutyrate and branched-chain fatty acids in the cecal digesta compared with the control chickens. Notably, dietary mangosteen peel preparations altered the antioxidant characteristics of the serum, liver, and thigh meat. Dietary MspE increased glutathione peroxidase (*P* = 0.039) in the serum and catalase in the serum (*P =* 0.008), liver (*P* = 0.05), and thigh meat (*P* = 0.01) compared to the control group. In addition, dietary MspP increased catalase levels in thigh meat compared to those in the control diet-fed chickens (*P* = 0.01). The concentration of malondialdehyde, an indicator of lipid peroxidation, was lower in all chicks-fed diets containing mangosteen peel preparations; however, statistical significance was only noted in the serum samples (*P* < 0.0001). Collectively, our study shows that dietary mangosteen peel preparations are potent natural antioxidants that can be used as functional dietary additives to effectively mitigate oxidative stress in broiler chicks.

## Introduction

Chicken meat is a major source of protein worldwide and its production capacity is projected to increase with the ever-increasing meat demand (Alexandratos and Bruinsma, 2012). This increase in meat demand was, in part, met by the addition of in-feed antibiotics to the diet of chickens, enabling rapid growth and disease prevention. However, owing to consumer concerns about antibiotic residues in poultry products (Roth et al., 2019), the use of in-feed antibiotics has been banned in the EU, and chickens are raised without in-feed antibiotics in most countries. However, microbial infections in poultry continue to negatively affect production by impairing digestive function, body weight, gut health, and meat quality (Abd El-Hack et al., 2021; Yaqoob et al., 2021). Hence, there has been growing interest in exploring alternative nutritional strategies to improve the performance and well-being of chickens (Windisch et al., 2008).

The potential of using botanicals has gained much interest as feed additives for livestock. These botanicals and their extracts are associated with beneficial effects on gut health, including stimulation of endogenous digestive enzymes, increased digestibility, and enhanced gut morphology (Jamroz et al., 2003; Mitsch et al., 2004; Diaz-Sanchez et al., 2015). In addition, these plant-derived products provide growth and health benefits owing to their antioxidant properties (Lee et al., 2004).

Mangosteen, also known as *Garcinia mangostana L*., is a tropical queen of fruits commonly found across India and Southeast Asia, and its peel extract has been used in herbal medicines (Suttirak and Manurakchinakorn, 2014). Mangosteen peels contain various phenolic compounds, including xanthones, benzophenones, flavonoids, bioflavonoids, phenols, and tannins (Suttirak and Manurakchinakorn, 2014; Manimekalai, 2016; Rohman et al., 2019). Xanthones are natural chemical substances classified as phenols or polyphenolic compounds (Adriani et al., 2020) and over 50 xanthones have been found in mangosteen (Akao et al., 2008). Xanthones have a six-carbon-conjugated ring structure with multiple double-carbon bonds. The most abundant xanthones in mangosteen are α-mangostin, β-mangostin, and γ-mangostin (Udaya Sankar et al., 2009; Rohman et al., 2019). α-Mangostin with antioxidant properties is the first xanthone isolated from mangosteen peel (Suttirak and Manurakchinakorn, 2014) and is the dominant component among the xanthones present in mangosteen (Wittenauer et al., 2012). Xanthones are insoluble in water (Rohman et al., 2019) and inhibit the de novo synthesis of cholesterol (Adriani et al., 2020). Furthermore, the mangosteen peel extract contains flavonoid compounds that diminish the absorption of cholesterol and bile acids in the small intestine, consequently contributing to a reduction in blood cholesterol levels (Adriani et al., 2020).

Mangosteen has been reported to exhibit various biological activities, including antioxidant, anti-allergic (Chae et al., 2012), anticancer (Jung et al., 2006; Akao et al., 2008; Mizushina et al., 2013), antitumor, and antibacterial (Gutierrez-Orozco and Failla, 2013). Recently, mangosteen products have been marketed as botanical dietary supplements in the United States because of their significant antioxidant potential (Jung et al., 2006), of which properties that are considered beneficial for poultry. Previous studies have been carried out using mangosteen peel powder and its extract for poultry nutrition. For example, Kusmayadi et al. (2019) found that 2% dietary mangosteen peel powder increased body weight and reduced serum cholesterol concentrations in ducks. Rusli et al. (2015) reported that dietary mangosteen peel powder at the level of 10 g/kg significantly lowered serum triglyceride levels in laying hens. Interestingly, the addition of mangosteen peel to the diet of broilers increased their body weight during heat stress (Hidanah et al., 2017). Furthermore, antimicrobial and antioxidant effects by dietary mangosteen peel extract were proved in broiler chickens infected with *Eimeria tenella* (Sriboonyong et al., 2022). The latter study found that the components in the mangosteen peel powder and its extract were not deposited in the meat or liver of the broilers (Sriboonyong et al., 2022) indicating that the putative components could exert their actions at the gut level. To the best of the authors' knowledge, the application of dietary mangosteen peel powder and its ethanolic extract was not tested for their efficacy in broiler chickens. Due to the strong antioxidant and antimicrobial activities, special emphasis was given to evaluate the role of dietary mangosteen peel powder and its ethanolic extracts in gut health and antioxidant properties in broiler chickens.

## Materials and methods

### Ethical statement

All chickens used in this experiment were cared for following protocols approved by the Institutional Animal Care and Use Committee at Konkuk University (KU23017).

### Birds, diets, and experimental design

A total 480 of 1-d-old straight-run broiler chicks (Ross 308) with similar body weights were randomly divided into four experimental treatments with eight replicates (12 birds each) and subjected to one of four experimental diets. A basal diet based on corn and soybean meal was formulated (Table 1). Experimental diets were formulated by mixing basal diet with mangosteen peel powder (MspP) at 20 g per kg or ethanolic extracts (MspE) at 0.5 g and 1 g per kg of diet. Cellulose was mixed with MspE to reach 20 g/kg of diet, and the control diet contained 20 g cellulose per kg of diet. Mangosteen peel preparations, powdered or ethanolic, were provided by Medi Bio Lab Co., Ltd. (Seoul 08389, Korea). The concentrations of α-mangostin in both powder and extracts were analyzed by high-performance liquid chromatography to contain 2.0% and 40.2% in the powdered and the extracts. The amounts of α-mangostin were calculated to contain 400 mg/kg in the diets added with the powdered form at 2% and the extracts at 0.1%, and 200 mg/kg in the diet added with the extract at 0.05%. The experiment lasted for 21 days. The chicks had *ad libitum* access to feed and water. The ambient temperature was set at 34°C on the first day of the experiment and gradually decreased to 24°C. Body weight and feed intake per pen were recorded on days 1 and 21 and were used to calculate the feed conversion ratio (FCR).

**Table 1:** Ingredients and chemical composition of the basal diet (%, as-fed basis).

Ingredients (g/100g)	CON^1^	MspP	MspE 0.05%	MspE 0.1%
Corn	56.46	56.46	56.46	56.46
Soybean meal, 44.7% CP	29.00	29.00	29.00	29.00
Corn gluten meal	7.00	7.00	7.00	7.00
Animal fat	2.00	2.00	2.00	2.00
Iodized salt	0.30	0.30	0.30	0.30
Monocalcium phosphate	1.30	1.30	1.30	1.30
_DL_-Methionine, 99%	0.35	0.35	0.35	0.35
_L_-Lysine-HCl, 56%	0.50	0.50	0.50	0.50
_L_-Threonine, 99%	0.10	0.10	0.10	0.10
Ground limestone	1.90	1.90	1.90	1.90
Sodium bicarbonate	0.24	0.24	0.24	0.24
Choline chloride, 50%	0.20	0.20	0.20	0.20
Vitamin premix^2^	0.20	0.20	0.20	0.20
Mineral premix^3^	0.25	0.25	0.25	0.25
Cellulose^4^	0.20	0.00	0.15	0.10
Mangosteen powder	-	0.20	-	-
Mangosteen ethanolic extract	0.00	-	0.05	0.10
Total	100.00	100.00	100.00	100.00
Calculated nutrient composition, %				
AMEn, kcal/kg	3,039	3,039	3,039	3,039
Dry matter	87.9	87.9	87.9	87.9
Crude protein	22.2	22.2	22.2	22.2
Calcium	1.02	1.02	1.02	1.02
Total phosphorus	0.71	0.71	0.71	0.71
Available phosphorus	0.45	0.45	0.45	0.45
Chloride	0.21	0.21	0.21	0.21
Sodium	0.21	0.21	0.21	0.21
Lysine	1.33	1.33	1.33	1.33
Methionine	0.72	0.72	0.72	0.72
Methionine + Cysteine	1.08	1.08	1.08	1.08
Threonine	0.92	0.92	0.92	0.92
Arginine	1.29	1.29	1.29	1.29
Histidine	0.55	0.55	0.55	0.55
Analzyed nutrient composition, %				
Dry matter	87.78	87.73	87.90	87.79
Crude protein	23.60	23.88	23.04	23.88
Crude fat	3.10	3.45	3.25	3.37
Crude fiber	4.01	3.34	4.07	2.99
Crude ash	8.57	8.33	8.74	8.39

AMEn, nitrogen-corrected apparent metabolizable energy.

1 CON, control diet; MspP; basal diet + 2% mangosteen peel powder; MspE 0.1% = basal diet + 0.1% mangosteen peel extract; MspE 0.05% = basal diet + 0.05% mangosteen peel extract.

2 Vitamin premix provided following nutrients per kg of diet: vitamin A, 9,000 IU; vitamin D_3_, 4,000 IU; vitamin E, 58 mg; vitamin K_3_, 2.7 mg; thiamine, 2.3 mg; riboflavin, 5.9 mg; vitamin B_5_, 17 mg; vitamin B_6_, 2.9 mg; vitamin B_12_, 0.015 mg; niacin, 54 mg; biotin, 0.16 mg; folate, 1.7 mg.

3 Mineral premix provided following nutrients per kg of diet: Fe, 57.1 mg; Mn, 85.7 mg; Zn, 64.3 mg; I, 0.57 mg; Se, 0.2 mg; Cu, 100 mg; Co, 0.17 mg.

4 Cellulose was mixed with mangosteen peel extracts to reach 20 g per kg of diet, whereas the control groups received 20 g cellulose per kg of diet.

### Sampling

At 21 d, one chicken per replicate was randomly selected for blood sampling after euthanasia using carbon dioxide. Immediately after blood sampling, the left leg (thigh meat and tibia), liver, ileum, and a pair of ceca were sampled and further processed on the day of sampling. The serum was separated by centrifugation at 200 × g for 15 min and stored at -20 ºC until analysis.

### Serum parameters including biochemical, immune, and antioxidant indices

Serum samples were analyzed using an automatic blood chemical analyzer (Film DRI CHEM 7000i, Fuji film, Tokyo, Japan) for total cholesterol (TCHO), triglycerides, high-density lipoprotein cholesterol (HDL-CHO), total protein, albumin, globulin, glutamic oxaloacetic transaminase (GOT), glutamic pyruvic transaminase (GPT), uric acid, and creatinine. The levels of Interferon-γ (IFR-γ) and interleukin-10 (IL-10) in the serum were determined using commercial ELISA kits (ThermoFisherScientific, Waltham, Massachusetts,USA). Alpha-1-acid glycoprotein ELISA kits (Abcam, Cambridge, MA, USA) were used to determine the level of alpha-1-acid glycoprotein in the serum, according to the manufacturer's instructions. Serum nitric oxide (NO) was measured using a modified Griess reagent (Sigma-Aldrich, St. Louis, MO, USA), and NO concentration was calculated from a standard curve with sodium titrate.

For antioxidant parameters in serum samples, various biomarkers of oxidative stress, including levels of glutathione peroxidase (GSH-Px; EnzyChrom glutathione peroxidase assay kit, BioAssay Systems, Hayward, CA, USA), total antioxidant capacity (TAC; QuantiChrom antioxidant assay kit, BioAssay Systems, Hayward, CA, USA) malondialdehyde (MDA; OxiSelect thiobarbituric acid reactive substances assay kit, Cell Biolabs, Inc, San Diego, CA, USA), superoxide dismutase (SOD; EnzyChrom superoxide dismutase assay kit, BioAssay Systems, Hayward, CA, USA), and catalase (CAT; OxiSelect catalase activity assay kit, Cell Biolabs, Inc, San Diego, CA, USA) were assayed. All assays were conducted using the corresponding kits, according to the manufacturer's instructions.

### Antioxidant capacity in liver and thigh meat

Approximately 1 g of liver and thigh meat were mixed with 9 ml of cold PBS and homogenized using Ultra Turrax (Digital Ultra-Turrax T25, IKA, Staufen, Germany). The homogenate was then centrifuged at 12,000 × *g* at 4 °C for 10 min, and the supernatant was stored at -20 °C until analysis. The diluted liver and meat supernatants were used for the determination of GSH-Px (EnzyChrom glutathione peroxidase assay kit, BioAssay Systems, Hayward, CA, USA), TAC (QuantiChrom antioxidant assay kit, BioAssay Systems, Hayward, CA, USA), superoxide dismutase (SOD; EnzyChrom superoxide dismutase assay kit, BioAssay Systems, Hayward, CA, USA), CAT (OxiSelect catalase activiti assay kit, Cell Biolabs, Inc, San Diego, CA, USA), MDA (OxiSelect thiobarbituric acid reactive substances assay kit, Cell Biolabs, Inc, San Diego, CA, USA) per the instructions described by the manufacturers. Results were normalized to the total protein concentration in each sample. Total protein concentrations in the liver and meat were quantified as described by Bradford (1976), using bovine serum albumin.

### Tibia characteristics

The tibia was obtained by removing attached meat and cartilage. The width and length of the tibia were measured using digital calipers. Tibia breaking strength was measured using an Instron (Model 3342, Instron Universal Testing Machine, Instron Corp., Norwood, MA, USA) with a 50 kg load range and a crosshead speed of 50 mm/min, with the tibia supported on a 3.35 cm span. Tibias were dried at 102 °C in drying oven for 24 h and weighed for dry matter determination. Dried tibias were extracted using a Soxhlet apparatus (Soxtherm automatic, Gerhardt, Bonn, Germany) for 24 h to measure the fat-free tibias. The fat-free tibias were then ashed at 600 °C for 3 h and reweighed.

### Meat quality

Leg meat was evaluated for cooking loss, pH, and color at 24 h postmortem. To measure cooking loss, fresh leg meat was placed in individual vacuum-sealed plastic bags, immersed in a water bath at 80 ºC for 30 min, and cooled in running tap water (in ice) for 20 min. The residual moisture was absorbed from each sample using tissue/filter papers. Cooking loss was calculated as the difference between the uncooked and cooked weights. The pH values of the breast and leg meats were measured at three locations using a portable pH meter (Testo 205, AG, Germany). The instrumental color of fresh meat, including lightness (L*), redness (a*), and yellowness (b*) values, was measured using a reflectance colorimeter (CM-2600d/2500d, Minolta, Japan). The color was measured in triplicate on the bone-side surface of each sample, and the colorimeter was calibrated throughout the measurement using a standard white ceramic tile.

### Short-chain fatty acid and branched-chain fatty acid in ceca digesta

Approximately 1 g of pooled cecal digesta was mixed with 9 ml of cold distilled water and homogenized using an Ultra Turrax (Digital Ultra-Turrax T25, IKA, Staufen, Germany). The mixture was added with 0.05 ml of saturated HgCl_2_, 1 ml of 25% H_3_PO_4,_ and 0.2 ml of 2% pivalic acid and centrifuged at 1,000 × g at 4 °C for 20 min. Then, the supernatant (1.5 ml) was collected and stored at -20 °C before analysis. The concentrations of short-chain fatty acids (SCFA) in the samples were measured using gas chromatography (6890 Series GC System, HP, Palo Alto, CA, USA), as described (Kim et al., 2020).

### Gut morphology

For histological examination, 1 cm-long ileal segment was collected from the middle of the ileum, fixed in 10% neutral-buffered formalin for 48 h, dehydrated, and embedded in a paraffin block. Histological sections (5 µm thick) were stained with hematoxylin and eosin using standard histological techniques. The mucosa was examined under a light microscope (Olympus BX43, Tokyo, Japan) and photographed using a digital camera (eXcope T500, DIXI Science, Daejeon, Korea). Ten intact, well-oriented villi and crypts were counted to determine villus height and crypt depth. Villus height was measured from the villus tip to the villus bottom, and crypt depth was defined from the villus bottom to the crypt. The ratio of villus height to crypt depth was calculated.

### Statistical analysis

All data were analyzed by analysis of variance in a completely randomized design using the general linear model procedure in SAS (SAS Institute Inc., Cary, NC, USA). All data were checked for outliers and normal distributions using the UNIVARIATE procedure in SAS (SAS Institute, Inc., USA). A pen was used as the experimental unit for growth performance while the individual bird was considered the experimental unit for the analysis of slaughtering parameters (i.e., tibia and meat qualities, villus morphology, cecal SCFA, antioxidant parameters in tissues, and serum parameters). The results are presented as least-squares means and pooled standard error of the mean. Tukey's honest significant difference test was used to determine the means and differences among treatments. Significant differences among treatments were determined at the probability of *P* ≤ 0.05, with 0.05 < *P* ≤ 0.10 considered a tendency.

## Results

None of the dietary treatments affected the production parameters (Table 2), meat and tibia characteristics (Table 3, Table 4), or ileal morphology (Table 5) of the broiler chicks. The concentrations of immune indicators (i.e., interferon-r, interleukin-10, alpha-1-acid glycoprotein, and nitric oxide) in serum samples were not altered by dietary treatments (Table 6). Dietary treatments did not affect serum biochemical parameters, except for the percentage of HDL-CHO, which was higher in chicks fed a diet containing 0.05% MspE than in the control group (Table 7).

**Table 2:** Effect of dietary mangosteen peel preparations on growth performance in broiler chickens^1^.

Item	CON^3^	MspP	MspE		SEM	*P*-value
			0.1%	0.05%		
Days 0 to 21						
BWG^2^, g/bird	744.9	711.0	715.6	709.5	10.005	0.118
FI, g/bird	1152.5	1105.7	1145.1	1128.7	12.187	0.219
FCR, g:g	1.549	1.561	1.602	1.591	0.017	0.272

1 Values are least squares means representing 8 observations.

2 BWG, body weight gain; FI, feed intake; FCR, feed conversion ratio.

3 CON, control diet; MspP, 2% mangosteen peel powder; 0.1% MspE = 0.1% mangosteen peel extract; 0.05% MspE = 0.05% mangosteen peel extract; SEM = standard errors of the means.

**Table 3:** Effect of dietary mangosteen peel preparations on thigh meat yield (g/100g of live body weight) and thigh meat quality in broiler chickens^1^.

Item	CON^2^	MspP	MspE		SEM	*P*-value
			0.1%	0.05%		
Thigh meat yield, g/ 100g of BW	6.455	6.737	5.994	6.442	0.239	0.488
Cooking loss, %	25.32	27.83	25.68	27.74	1.090	0.431
pH	5.782	5.770	5.739	5.760	0.023	0.736
L* (lightness)	56.67	57.47	56.60	57.82	0.569	0.587
a* (redness)	7.924	7.923	8.080	7.676	0.255	0.885
b* (yellowness)	20.11	20.47	20.70	20.75	0.393	0.747

1 Values are least squares means representing 8 observations.

2 CON, control diet; MspP, 2% mangosteen peel powder; 0.1% MspE, 0.1% mangosteen peel extract; 0.05% MspE, 0.05% mangosteen peel extract; SEM = standard errors of the means.

**Table 4:** Effect of dietary mangosteen peel preparations on tibia characteristics in broiler chickens^1^.

Item^2^	CON^3^	MspP	MspE		SEM	*P*-value
			0.1%	0.05%		
Fresh tibia weight, g/100 g of live BW	0.783	0.796	0.786	0.795	0.034	0.995
Length, cm	7.544	7.548	7.495	7.388	0.067	0.563
Width, cm	0.559	0.530	0.531	0.543	0.010	0.242
Strength, kgf	15.92	14.33	15.41	15.03	0.585	0.461
Dry matter, %	36.71	36.34	36.99	37.59	0.373	0.385
Ash/fat-free dry matter, %	43.96	42.85	42.93	43.53	0.559	0.614

1 Values are least squares means representing 8 observations.

2 BW, body weight.

3 CON, control diet; MspP, 2% mangosteen peel powder; 0.1% MspE, 0.1% mangosteen peel extract; 0.05% MspE, 0.05% mangosteen peel extract; SEM = standard errors of the means.

**Table 5:** Effect of dietary mangosteen peel preparations on ileal morphology in broiler chickens^1^.

Item^2^	CON^3^	MspP	MspE		SEM	*P*-value
			0.1%	0.05%		
Villus height, µm	760.7	685.2	752.4	683.7	23.558	0.150
Crypt depth, µm	140.0	129.4	135.6	122.7	4.722	0.198
VH to CD ratio	5.539	5.412	5.740	5.600	0.290	0.956

1 Values are least squares means representing 8 observations.

2 VH, villus height; CD, crypt depth.

3 CON, control diet; MspP, 2% mangosteen peel powder; 0.1% MspE, 0.1% mangosteen peel extract; 0.05% MspE, 0.05% mangosteen peel extract; SEM = standard errors of the means.

**Table 6:** Effect of dietary mangosteen peel preparations on innate immunity markers in serum of broiler chickens^1^.

Item^2^	CON^3^	MspP	MspE		SEM	*P*-value
			0.1%	0.05%		
Interferon-γ, pg/ml	1.693	1.721	1.640	1.728	0.052	0.827
Interleukin-10, pg/mL	79.17	75.20	71.36	76.38	3.677	0.686
Alpha-1-acid glycoprotein, µg/mL	190.4	200.3	202.6	230.7	11.182	0.265
Nitric oxide, µM	29.16	31.47	23.11	25.85	2.981	0.477

1 Values are least squares means representing 8 observations.

2 IFR-γ, interferon- γ; IL-10, interleukin-10.

3 CON, control diet; MspP, 2% mangosteen peel powder; 0.1% MspE, 0.1% mangosteen peel extract; 0.05% MspE, 0.05% mangosteen peel extract; SEM = standard errors of the means.

**Table 7:** Effect of dietary mangosteen peel preparations on serum biological parameters in broiler chickens^1^.

Item^2^	CON^3^	MspP	MspE		SEM	*P*-value
			0.1%	0.05%		
TCHO, mg/dL	124.1	121.3	119.0	124.0	4.769	0.934
Triglyceride, mg/dL	69.31	64.50	53.38	53.38	5.376	0.228
HDL-CHO, mg/dL	90.36	101.38	96.50	109.57	4.562	0.117
HDL-CHO, % of TCHO	72.83^b^	83.83^a^	81.01^ab^	87.93^a^	2.338	0.004
Total protein, mg/dL	2.431	2.288	2.138	2.575	0.142	0.443
Albumin, mg/dL	0.875	0.913	0.863	0.950	0.041	0.678
Globulin, mg/dL	405.5	372.3	350.6	369.4	14.875	0.173
GOT, U/L	142.8	146.4	139.3	149.9	6.214	0.443
GPT, U/L	31.38	32.25	32.88	30.00	0.791	0.305
Uric acid, mg/dL	12.58	11.11	11.09	10.23	0.819	0.376
Creatinine, mg/dL	0.663	0.538	0.563	0.600	0.054	0.527

1 Values are least squares means representing 8 observations.

2 TCHO, total cholesterol; HDL-CHO, high-density lipoprotein cholesterol; GOT, glutamic oxaloacetic transaminase; GPT, glutamic pyruvic transaminase.

3 CON, control diet; MspP, 2% mangosteen peel powder; 0.1% MspE, 0.1% mangosteen peel extract; 0.05% MspE, 0.05% mangosteen peel extract; SEM = standard errors of the means.

Indicators of antioxidant capacity were monitored in serum, liver, and thigh meat samples (Table 8). SOD activity and TAC in the serum samples were not affected by the dietary treatments. However, GSH-Px activity increased (*P* = 0.039) in the 0.1% MspE group compared to that in the control group. Serum CAT levels were higher (*P* = 0.008) in the 0.05% MspE group than those in the control group. MDA levels in serum samples were lower (*P* < 0.001) in mangosteen peel-fed chickens than those in the control group. GSP-Px activity in the liver was highest in chicks fed a diet containing 0.05% MspE but was not statistically different from that in the control group. Dietary MspE at 0.05% increased CAT activity in the liver samples compared to that in the control group (*P* = 0.05). The MDA level tended to be low (*P* = 0.052) by on average 9.2 to 20.2% in mangosteen peel-fed vs. the control diet-fed groups. However, none of the dietary treatments affected SOD activity in liver. TAC levels in the liver tended to be higher in all treated groups compared with control group (*P* = 0.088). CAT activity in thigh meat was significantly elevated in chicks fed diets containing MspP and 0.1% MspE compared to the control group (*P* = 0.01). Other antioxidant indices in thigh meats were not affected (*P* > 0.05) by the dietary treatments. However, all treatment groups tended to increase TAC levels in the thight meats compared to the control group (*P* = 0.095).

**Table 8:** Effect of dietary mangosteen peel preparations on oxidative stress markers in serum, liver, and thigh meats of broiler chickens^1^.

Item^2^	CON^3^	MspP	MspE		SEM	*P*-value
			0.1%	0.05%		
**Serum samples**						
GSH-Px activity, U/L	435.7^b^	442.2^b^	541.4^a^	524.7^ab^	24.964	0.039
SOD activity, U/L	0.0985	0.0981	0.0986	0.0994	0.001	0.733
CAT, U/mL	5.04^b^	10.74^ab^	13.12^ab^	17.84^a^	2.146	0.008
MDA, µM	32.97^a^	18.81^b^	15.71^b^	19.73^b^	1.804	<.0001
TAC, mM	0.854	1.030	0.958	0.902	0.050	0.105
**Liver samples**						
GSH-Px activity, U/mg of protein	93.7^ab^	88.1^b^	96.6^ab^	101.9^a^	2.867	0.039
SOD activity, U/mg of protein	0.119	0.120	0.117	0.121	0.001	0.639
CAT, U/mg of protein	87.3^b^	97.0^ab^	101.2^ab^	119.6^a^	6.878	0.050
MDA, nmol/mg of protein	1.205	1.053	0.961	1.094	0.057	0.052
TAC, nmol/mg of protein	68.29	73.85	69.91	71.80	1.491	0.088
**Thigh meat samples**						
GSH-Px activity, U/mg of protein	5.064	5.706	5.794	5.383	0.588	0.830
SOD activity, U/mg of protein	0.0067	0.0072	0.0070	0.0078	0.0003	0.216
CAT, U/mg of protein	5.714^b^	9.600^a^	10.115^a^	7.441^ab^	0.867	0.010
MDA, nmol/mg of protein	1.890	1.419	1.247	1.402	0.204	0.168
TAC, nmol/mg of protein	33.37	35.47	39.33	39.08	1.735	0.095

1 Values are least squares means representing 8 observations.

2 GSH-Px, glutathione peroxidase; SOD, superoxide dismutase; CAT, catalase; MDA, malondialdehyde; TAC, total antioxidant capacity.

3 CON, control diet; MspP, 2% mangosteen peel powder; 0.1% MspE, 0.1% mangosteen peel extract; 0.05% MspE, 0.05% mangosteen peel extract; SEM, standard errors of the means.

The absolute and relative concentrations of short fatty acids in the cecal digesta are shown in Table 9. Dietary treatments did not affect the absolute concentrations of short fatty acids but affected the relative concentrations of branched fatty acids in the cecal digesta. Chicks fed the MspP-supplemented diet exhibited the lowest concentrations of isobutyrate (*P* = 0.049) and branched-chain fatty acids (*P* = 0.034) compared to the control group. And all treatment groups showed a tendency for decreased concentration of valerate compared to the control group (P = 0.078). There were no apparent differences in the absolute and relative concentrations of acetate, propionate, or butyrate in the cecal digesta (*P* > 0.05).

**Table 9:** Effect of dietary mangosteen peel preparations on the absolute or relative concentrations (mmol/kg digesta, % of total) of cecal short- and branched-chain fatty acids in broiler chickens^1^.

Item^2^	CON^3^	MspP	MspE		SEM	*P*-value
			0.1%	0.05%		
**mmol/kg**						
Acetate	71.11	94.66	76.40	82.68	7.801	0.415
Propionate	1.729	2.228	1.007	1.373	0.326	0.292
Isobutyrate	0.722	0.426	0.739	0.491	0.094	0.191
Butyrate	21.03	28.11	14.85	18.31	3.197	0.234
Isovalerate	0.826	0.664	0.716	0.460	0.145	0.511
Valerate	1.299	1.351	1.193	1.131	0.150	0.862
BCFA	2.847	2.441	2.648	2.082	0.318	0.537
Total SCFA	96.72	127.44	94.90	104.44	10.290	0.383
**% of total SCFA**						
Acetate	74.72	75.01	80.31	78.59	2.002	0.322
Propionate	1.612	1.637	1.029	1.279	0.191	0.255
Isobutyrate	0.768^a^	0.338^b^	0.842^a^	0.481^ab^	0.103	0.049
Butyrate	20.68	21.46	15.74	18.10	1.816	0.325
Isovalerate	0.850	0.501	0.856	0.464	0.132	0.200
Valerate	1.373	1.052	1.222	1.086	0.078	0.078
BCFA	2.992^a^	1.890^b^	2.920^ab^	2.031^ab^	0.250	0.034

1 Values are least squares means representing 8 observations.

2 SCFA, short-chain fatty acids (acetate + propionate + butyrate + isobutyrate + isovalerate + valerate); BCFA, branched-chain fatty acids (isobutyrate + valerate + isovalerate).

3 CON, control diet; MspP, 2% mangosteen peel powder; 0.1% MspE, 0.1% mangosteen peel extract; 0.05% MspE, 0.05% mangosteen peel extract; SEM, standard errors of the means.

## Discussion

Owing to the biological properties (i.e., antimicrobial, immune-modulating, and antioxidant) of mangosteen peel (Jung et al., 2006; Ele et al., 2018; Sriboonyong et al., 2022), it is expected to have beneficial effects on broiler chicks fed diets containing either MspP or MspE. In contrast to our expectations, dietary mangosteen peel preparations in powdered form or their ethanolic extracts did not affect the growth performance and meat and tibia quality in broiler chicks. This finding is in agreement with earlier studies that showed that mangosteen peel or its extracts had no significant impact on chicken performance (Herawati et al., 2020). However, dietary mangosteen peel increased the body weight of chickens exposed to high ambient temperatures compared to the heat-stressed control group (Hidanah et al., 2017). Thus, it is likely that mangosteen peel influences the performance of chickens in suboptimal environments, including heat stress or pathogen-mediated immune suppression. Whether dietary mangosteen peel preparations can relieve stress (i.e., heat stress or pathogen challenge)-induced decrease in chicken performance needs to be addressed.

As mangosteen peels have immunomodulatory and antimicrobial activities (Ele et al., 2018; Sriboonyong et al., 2022), we next attempted to measure the markers of innate immunity and gut health in chickens. Cytokines are major factors involved in the communication between T cells, macrophages, and other immune cells in the immune response to antigens and infectious agents (Ferro et al., 2004). Alpha-1-acid glycoprotein, a predominant acute-phase protein in avians, plays a significant role in defense mechanisms by combating infectious microbes, facilitating tissue repair, and promoting overall health (Eckersall and Bell, 2010). Alpha-1-acid glycoprotein has been used as a marker to assess non-specific systemic inflammation in chickens (Lee et al., 2017). Nitric oxide that is produced by chicken monocyte and macrophage upon exposure to bacteria was assayed as an added measure of immune status of chickens (Lee et al., 2011). None of dietary treatments affected the markers of innate immunity including interferon-r, interleukin-10, alpha-1-acid glycoprotein and nitric oxide in serum samples of chickens. It is speculated that the lack of mangosteen peel preparations on innate immunity might be in part related to the use of naïve chickens. Thus, the effect of mangosteen peel preparations in challenged chicken models including lipopolysaccharide, *Eimeria* infection or necrotic enteritis disease would be more relevant to see their beneficial effect, if any, that needs to be answered.

As the indicators of gut health, ileal morphology and cecal volatile fatty acids were measured (Kim et al., 2024). However, ileal morphology, including villus height, crypt depth, and their ratios, was not affected by the dietary treatments. Our findings agree with that of a previous study by Kim et al. (2023) who reported that dietary MspE (5 g/kg of diet) did not affect villus length and crypt depth in pigs. Although dietary mangosteen peel preparations did not affect ileal morphology, they lowered the relative concentrations of isobutyrate and branched-chain fatty acids in the cecal digesta compared with the control group. Thus, it is likely that dietary MspP, but not MspE, affects the cecal bacterial community, which uses undigested proteins as the preferential substrate in the ceca, leading to a reduction in branched-chain fatty acids. However, it is not clearly understood how MspP is more effective than MspE in modulating cecal-branched fatty acids. At this stage, no clear explanation is available. It can be postulated that the unknown components present in mangosteen peel, but not in its ethanolic extract, would affect the concentrations of branched fatty acids. To clearly address this, further study is needed to elucidate the role of extracts and residues of mangosteen peel on the gut microbiome and their metabolites including cecal volatile fatty acids.

Because of the cholesterol-lowering effects of mangosteen peels (Hidanah et al., 2017; Kusmayadi et al., 2019), we attempted to measure cholesterol levels in serum samples. Although dietary mangosteen peel preparations did not affect lipid metabolism (as manifested by the lack of difference in the concentrations of total cholesterol, HDL cholesterol, and triglycerides in serum samples), the relative percentage of HDL cholesterol was elevated in all treated diet-fed chicks, especially in those fed diets supplemented with MspP, and 0.05% MspE was significantly higher than that in the control diet-fed chicks. Thus, our study partially confirms previous studies showing the hypocholesterolemic properties of mangosteen fruit and peel preparations (Chomnawang et al., 2007; Adiputro et al., 2013). Other than lipid profiles, serum biochemical profiles, including GOT, GPT, total protein, albumin, globulin, uric acid, and creatinine levels, were not altered by the dietary treatments. Thus, the dietary mangosteen peel preparations used in this study did not negatively affect the hepatic or kidney function. Altered concentrations of GOT and GPT in the blood are considered pathological alterations in the liver (Kim et al., 2018).

Several studies have reported that a mangosteen-based diet has antioxidant properties (Chae et al., 2012; Sriboonyong et al., 2022; Ruankham et al., 2022) which decided to measure the biological markers for enzymatic and non-enzymatic antioxidant systems. Oxidative stress occurs when the balance between the antioxidant defense system and the free radical generation system in animals is disturbed, leading to several diseases (Miguel et al., 2009; Masood et al., 2013; Bai et al., 2017; Kikusato, 2021; Mahfuz et al., 2021; Sierżant et al., 2023). Broiler chicks are susceptible to oxidative damage owing to their physiological characteristics (Bai et al., 2017). Thus, an array of biological markers of enzymatic/non-enzymatic antioxidant systems and lipid peroxidation in chickens was assayed. In this study, dietary mangosteen peel preparations increased GSH-Px and CAT levels in serum and liver samples, and CAT in thigh meat, compared with the control chickens. Consequently, MDA concentrations were kept significantly low (*P* < 0.001) in serum samples and marginally low in the liver (*P* = 0.052) and thigh meat (*P* = 0.168) from the mangosteen peel preparations-fed vs. the control diet-fed chickens. Our study clearly shows that mangosteen peel preparations possess antioxidant activity, which activates enzymatic antioxidant systems and retards lipid peroxidation in broiler chickens. Among the components present in mangosteen peel preparations, α-mangostin is the active and dominant one that is known to be a potent antioxidant (Masood et al., 2013; Suttirak and Manurakchinakorn, 2014; Sriboonyong et al., 2022; Ruankham et al., 2022). In a previous study, the antioxidant effect of mangosteen peel was found to be more potent than that of ascorbic acid and Trolox (Sriboonyong et al., 2022). The antioxidant activities of mangosteen peel preparations (either powdered or extracted) have been reported in rats and mice (Samuagam et al., 2015; Elmund and Hartrianti, 2020). To the best of our knowledge, this is the first study to show that dietary mangosteen peel preparations act as natural antioxidants that are systemically active upon ingestion by broiler chicks. Previous studies have demonstrated that natural polyphenol compounds in plants can activate the nuclear factor erythroid 2-related factor 2 (Nrf2) pathway in cells, which then induces the expression of ROS scavengers, such as SOD and CAT (Kikusato, 2021). Nrf2 is known to be a key transcription factor that regulates the expression of phase II metabolic enzymes (i.e., glutathione-related enzymes, CAT, and SOD) and proteins that protect host cells against oxidative damage triggered by injury and inflammation (Kim et al., 2010). However, this study did not investigate the effects of dietary mangosteen peel preparations on the gene expression of antioxidant enzymes. Thus, an in-depth study on the effects of dietary mangosteen peels on broiler antioxidant systems is warranted.

It should also be remembered that the effect of MspE at 0.05% and 0.1% was not exhibited dose-dependant on all antioxidant parameters assayed in this study. For example, hepatic GSH-Px activity was numerically higher in 0.05% MspE-added diet-fed broilers compared with the 0.1% MspE-fed counterparts. This trend (elevated activity by low vs. high MspE) was also found in serum CAT levels. These findings might not indicate the negative effect of higher MspE levels as it did not impair production performance nor affect the indicators of liver and kindey functions (e.g., GOT and GPT). Instead, our study clearly shows that low level of MspE at 0.05% is equally effective in augmenting the systemic antioxidant functions.

In conclusion, neither mangosteen peel powder nor its ethanolic extract affected growth performance, meat quality, ileum morphology, or immune parameters in broiler chicks. However, they influenced the percentage of HDL cholesterol and altered antioxidant markers in the serum, liver, and thigh meat. Collectively, our study confirmed the potent antioxidant activities of mangosteen peel powder and its ethanolic extracts in broiler chickens. Additional studies are currently ongoing to elucidate whether dietary mangosteen peel preparations would affect the performance and health of broiler chickens exposed to pathogens (e.g., *Eimeria* spp.).

## Declaration of competing interest

No potential conflict of interest was reported by the authors.
